# Thyroid-Induced Worsening of Parkinsonian Tremor Resistant to Drugs and Subthalamic Nucleus Deep Brain Stimulation

**DOI:** 10.1155/2014/489275

**Published:** 2014-12-31

**Authors:** Michal Minár, Peter Valkovič

**Affiliations:** ^1^Second Department of Neurology, Faculty of Medicine, Comenius University, Limbová 5, 83305 Bratislava, Slovakia; ^2^Laboratory of Motor Control, Institute of Normal and Pathological Physiology, Slovak Academy of Sciences, Sienkiewiczova 1, 813 71 Bratislava, Slovakia

## Abstract

*Introduction.* Symptoms of both hypothyroidism and thyrotoxicosis can be easily overlooked in patients with Parkinson's disease (PD). We report on a patient whose parkinsonian tremor worsened and proved refractory not only to common treatment, but also to deep brain stimulation (DBS). *Case Presentation.* A 61-year-old woman with advanced PD underwent bilateral subthalamic DBS, with an excellent outcome. Twenty-one months after the surgery, however, patient's resting/postural tremor markedly worsened. There was a slight improvement for 1 month after repeated adjustments of DBS parameters, but then the tremor worsened again. Since even a minimal increase of the dose of dopaminergic drugs caused extremely severe dyskinesias, an anticholinergic drug biperiden and benzodiazepine clonazepam were introduced, what helped for another month. With the onset of severe diarrhoea, a laboratory workup was performed. Thyrotoxicosis was detected. During treatment with the antithyroid agent carbimazole, the parkinsonian tremor clearly improved within two weeks. *Conclusion.* A hyperthyroid state can markedly exaggerate all forms of tremor, as well as other types of movement disorders. This condition can be overlooked or masked by other symptoms. Therefore, if the tremor in a patient with PD gradually worsens and proves resistant to the usual treatment, examine the thyroid gland.

## 1. Introduction

Symptoms of hypothyroidism, for example, locomotor slowness and hypomimia, can be easily overlooked in patients with Parkinson's disease (PD), especially in the elderly. On the other hand, another sign of PD tremor is also seen in hyperthyroid state. For these reasons, some authors have looked for a link between PD and abnormalities of the thyroid gland with contradictory results. There is a disagreement, whether hypothyroidism is or is not more prevalent in PD patients [1]. Nevertheless, since parkinsonian tremor is known to be exaggerated by thyrotoxicosis [2, 3], it was assumed that the hyperthyroid state leads to increased catabolism of dopamine in the CNS, resulting in a need for higher doses of antiparkinsonian drugs. But the hyperthyroid state causes enhanced tremor not only in PD, but also in essential tremor [2], bilateral chorea-ballism [4], and tremor in Wilson's disease (not caused by dopamine depletion).

We report on a patient whose parkinsonian tremor worsened and proved refractory not only to dopaminergic and anticholinergic treatment, but also to bilateral subthalamic deep brain stimulation. Thyrotoxicosis was the hidden cause.

## 2. Case Report

A 61-year-old woman had been diagnosed 16 years previously to have Parkinson's disease of equivalent type with asymmetrical rigidity, hypokinesia, resting tremor, and mild postural instability. Because of the progressive worsening of motor and nonmotor fluctuations, resistant to oral medication adjustments, we decided to go for surgical treatment. After successful bilateral subthalamic deep brain stimulation (DBS), she enjoyed an excellent outcome. Twenty-one months after the surgery, however, her asymmetrical resting/postural tremor, particularly affecting the lower extremities, markedly worsened (from 1 to 3; score according to the Unified PD Rating Scale, part III, item 20). After a short episode of benign gastrointestinal “virosis,” the worsening persisted, and the tremor spread to all four extremities. There was a slight improvement for 1 month after repeated adjustments of DBS parameters (all: voltage, pulse width, and frequency), but then the tremor worsened again. Since even a minimal increase of the dose of levodopa and/or pramipexole caused extremely severe dyskinesias, an anticholinergic drug biperiden (2 mg a day) and benzodiazepine clonazepam (2 mg a day) were introduced. This approach helped for another month, but biperiden led to increased confusion. The patient lost 15 kilograms in weight over 6 months. The cause of weight lost was assumed most likely due to adverse effects of the drugs (loss of appetite due to levodopa, dopamine agonist, and anticholinergic agent) combined with increased energy consumption due to the dyskinesias and tremor. With the onset of severe diarrhoea (8–10 times a day), an internist was consulted and a laboratory workup was performed. Thyrotoxicosis was detected (TSH < 0.01 mlU/L (normal range: 0.4–4.0 mlU/L), fT3 = 13.3 pmol/L (normal range: 3.0–6.5 pmol/L), and fT4 = 48.3 pmol/L (normal range: 9.0–23.0 pmol/L)). During treatment with the antithyroid agent carbimazole (12 mg a day), the parkinsonian tremor clearly improved within 2 weeks. The patient gained 12 kilograms over the following 2 months and now feels well again.

## 3. Discussion

Tremor consists of involuntary rhythmic movements, oscillations, of one or more parts of the body, depending on the mechanical properties of tissues and central neurogenic oscillations. There are several central generators of oscillation in the nervous system, for example, the cerebellothalamic loop or circuits of the basal ganglia (BG). As all of these nuclei are interconnected, their oscillatory activity may converge at the level of the thalamic ventral nuclei [2]. Tremor is observed in the healthy, but it can also be a symptom of disease.

Both the BG and cerebellothalamocortical (CTC) circuits are responsible for parkinsonian resting tremor. The depletion of pallidal dopamine (due to a lack of projection from the substantia nigra and retrorubral area) leads to excessive synchronization of neurons and thus to enhanced connectivity between the BG and CTC circuits, which exacerbates the tremor activity (the BG are only a trigger) [5]. The cerebellum moderates this activity that is thought to originate in the ventrolateral thalamus with its connection to the subthalamic nucleus and zona incerta. Since the motor cortex is the only part directly projecting to the spinal motor neurons, it is the site of voluntary movements and probably drives the tremor in PD; however, these two activities do not occur at the same time in PD patients. It has been hypothesized that parkinsonian tremor could be (at least partially) an attempt of the motor cortex to compensate for the hypokinetic state by facilitating movement initiation in hyperexcited cortical circuits. This theory is supported by the following facts: (i) tremor is less responsive to dopaminergic treatment, (ii) it is more obvious in earlier stages of PD and in patients with tremor-dominant PD (here the CTC circuits are not yet damaged and are able to produce tremor), and (iii) tremor occurs a few days after hypokinesia and rigidity in animal models of PD [6].

Thyroid hormones (thyroxine and triiodothyronine) increase the expression of calcium currents as well as beta-adrenergic receptors connected with calcium channels in all excitable cells [7], including the central and peripheral nervous systems. The enhanced neuronal excitability or hypersensitivity in the oscillatory loops possibly exacerbates the tremor. The following facts are well known: first, tremor worsens with stress, emotions, hypoglycaemia, and some stimulating drugs (all hyperadrenergic states); second, symptoms are mitigated with beta-blockers, for example, propranolol. Beta-blockers have an immediate effect since they antagonize the adrenergic receptors, whereas antithyroid drugs act more slowly by reducing beta-receptors after thyroid hormone blood levels fall.

## 4. Conclusion

A hyperthyroid state can markedly exaggerate all forms of tremor as well as other types of movement disorders. This condition can be masked by other symptoms or simply be overlooked. Therefore, if the tremor in a PD patient gradually worsens and proves to be resistant to the usual antiparkinsonian (or antitremor) treatment, examine the thyroid gland.
